# Supplementary material for: An Innovative Test for the Rapid Detection of Specific IgG Antibodies in Human Whole-Blood for the Diagnosis of Opisthorchis viverrini Infection
Source: Trop Med Infect Dis. 2022 Oct 17;7(10):308. doi: 10.3390/tropicalmed7100308 (PMC9607866; doi:10.3390/tropicalmed7100308)
Supplement: Supplementary file 1 [file tropicalmed-07-00308-s001.zip › tropicalmed-1946492-supplementary.pdf]

Tropical Medicine and Infectious Disease

MDPI

Supplementary Materials for An Innovative Test for the Rapid Detection of Specific IgG Antibodies in Human Whole-Blood for the Diagnosis of *Opisthorchis viverrini* Infection

Table S1. Evaluation of the OvB-test kit using the EDTA anti-coagulated whole blood samples from the field.

No.	Parasitosis*	EPG**	Level in OvB-test kit****
1	Opisthorchiasis	18	<2
2	Opisthorchiasis	68	0.5
3	Opisthorchiasis	16	0.5
4	Opisthorchiasis	10	0.5
5	Opisthorchiasis	23	<3
6	Opisthorchiasis	44	0.5
7	Opisthorchiasis	26	0.5
8	Opisthorchiasis	12	2
9	Opisthorchiasis	38	1
10	Opisthorchiasis	21	0.5
11	Non-infected individual	0	Negative
12	Non-infected individual	0	Negative
13	Non-infected individual	0	Negative
14	Non-infected individual	0	Negative
15	Non-infected individual	0	Negative
16	Non-infected individual	0	Negative
17	Non-infected individual	0	Negative
18	Non-infected individual	0	Negative
19	Non-infected individual	0	Negative
20	Non-infected individual	0	Negative
21	Blastocystosis	0	Negative
22	Blastocystosis	0	Negative
23	*Entamoeba coli* infection	0	Negative
24	*Entamoeba coli* infection and strongyloidiasis	0	Negative
25	Lecithodendriid fluke infections	14***	Negative
26	Taeniasis	0	Negative
27	Hymenolepiasis (*Hymenolepis diminuta*)	136	Negative
28	Hymenolepiasis (*Hymenolepis nana*)	20	Negative
29	Blastocystosis and lecithodendriid fluke infections	14***	0.5
30	Sarcocystosis and Blastocystosis	0	Negative
31	Strongyloidiasis	0	Negative
32	Strongyloidiasis	0	Negative
33	Strongyloidiasis	0	0.5
34	Strongyloidiasis	0	Negative
35	Strongyloidiasis	0	Negative
36	Strongyloidiasis	0	Negative
37	Strongyloidiasis	0	Negative
38	Strongyloidiasis	0	Negative
39	Strongyloidiasis	0	Negative
40	Strongyloidiasis	0	0.5

*Diagnosed by the modified formalin ethyl acetate concentration method [1]. ** Parasite egg count (EPG). *** lecithodendriid fluke (EPG). **** Color band intensity (cut-off ≥ 0.5). No. 31-40, ten strongyloidiasis cases were used to evaluate due to high incidence infection in Khon Kaen Province.

Reference

1. Elkins, D.B.; Haswell-Elkins, M.; Anderson, R.M. The epidemiology and control of intestinal helminths in the Pulicat Lake region of Southern India. I. Study design and pre- and post-treatment observations on Ascaris lumbricoides infection. *Trans. R. Soc. Trop. Med. Hyg.* **1986**, *80*, 774–792. https://doi.org/10.1016/0035-9203(86)90384-6.
